# Contrast-enhanced ultrasonographic imaging of hepatic splenosis: A case report

**DOI:** 10.1097/MD.0000000000024243

**Published:** 2021-01-22

**Authors:** Xiaofei Zhong, Lulu Yang, Jiayan Huang, Liping Deng, Ling Nie, Qiang Lu

**Affiliations:** a Department of Ultrasound; b Department of Radiology; c Department of Pathology, West China Hospital, Sichuan University, Chengdu, Sichuan Province, P.R. China.

**Keywords:** contrast-enhanced ultrasound, diagnosis, splenosis

## Abstract

**Rationale:**

Hepatic splenosis or heterotopic auto-transplantation of spleen in the liver usually occurs after either spleen trauma or surgery. It is of great importance for the differential diagnosis of hepatic splenosis and other liver tumors because surgery is usually not needed if a diagnosis of splenosis is confirmed.

**Patient concerns:**

Multiple hepatic masses were revealed by grayscale ultrasound in a 55-year-old man complaining of persistent colic in the upper abdomen after greasy food.

**Diagnosis:**

Benign neoplasm with enlarged lymph node in the gastro-hepatic ligament was suspected by contrast enhanced US. The nature of the hepatic mass was undetermined by CECT.

**Interventions:**

The lesions were surgically removed.

**Outcomes:**

Multiple splenic tissue implants in the liver and peritoneum were confirmed by pathology after surgery. The patient recovered well and was followed up for more than 1 year without recurrence.

**Lessons:**

Splenosis should be included in the differential diagnosis of focal liver lesion in patients with a history of spleen trauma or surgery. In spite of nonspecific findings on pre-contrast ultrasound, splenosis shows characteristic homogeneous hyperenhancement in arterial and portal phases, as well as prolonged hyperenhancement in the late phase for more than 5 minutes. Furthermore, the confidence of the diagnosis of splenosis may be enhanced by identifying multiple masses with similar enhancing patterns in other regions of the abdominal cavity.

## 1 Introduction

Splenosis refers to ectopic implantation of splenic tissue after splenic trauma or surgery. The implants are usually incidental asymptomatic findings. Because of the compensatory growth of splenic tissue implantation, it is beneficial to the recovery of splenic function after splenectomy.^[1]^ However, there may also be necrosis, adhesive ileus, abdominal abscess, and other complications.^[2]^ No clinical symptoms of splenosis can be followed up.^[3]^

In recent years, a number of studies have reported the radiological findings of splenosis, including computer tomography (CT), magnetic resonance imaging, ultrasonography and radionuclide scintigraphy. Contrast-enhanced (CE) CT revealed uniformly enhanced splenosis in the arterial and portal venous phases.^[4]^ After absorption of superparamagnetic iron oxide, the signal intensity of T2WI decreased.^[5]^ S. Greschus reported a case of splenosis diagnosed by erythrocyte scintigraphy, in which Tc-99m labeled heat-denatured erythrocytes accumulated in the lesion.^[6]^

Although the incidence of hepatic splenosis is rare, it is of great importance for the differential diagnosis of this pathologic entity and other liver tumors, because surgery is usually not needed if a diagnosis of splenosis is confirmed. Herein, we report a case of pathologically proven hepatic splenosis that was initially diagnosed as liver tumor.

## 2 Case report

A 55-year-old man visited the hospital for persistent cramps in the upper abdomen after greasy food 20 days prior. The pain was relieved with prone position or by pressing the upper abdomen. The patient had no chills, fever, sweating, or jaundice. The patient had a history of spleen excision 30 years ago due to abdominal trauma. The patient denied any history of hepatitis. Serum laboratory tests displayed the following results: total bilirubin 28.9 μmol/L (reference value (RF) 5.0–28.0 μmol/L), direct bilirubin 11.8 μmol/L (RF < 8.8 μmol/L), alanine aminotransferase 132 IU/L (RF <50 IU/L), aspartate aminotransferase 57 IU/L (RF < 40 IU/L), total protein 56.6 g/L (RF: 65.0–85.0 g/L), albumin 34.9 g/L (RF: 40.0–55.0 g/L), serum carbohydrate antigen 19 to 9 22.9 IU/ ML (RF < 22 g/L) and hepatitis B surface antibody 354.800 IU/L (+).

### 2.1 Imaging findings

The patient received routine grayscale US scanning that revealed 3 focal liver lesions (FLL), and further CE CT and CEUS were performed to determine the nature of the lesions. The findings of CECT and CEUS are described below.

#### 2.1.1 CECT imaging

**Figure 1:**
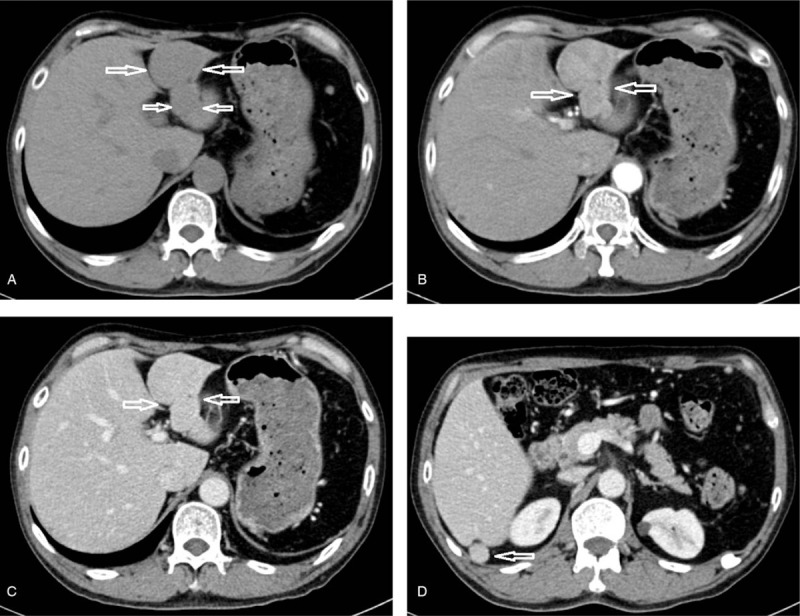
Computer tomography scan of a 55 year old man complaining of upper abdominal pain. A: A slightly lobulated low density lesion measuring 4.0 x 3.7 cm (white arrow) was demonstrated adjoining to the ligamentum teres on pre-contrast computer tomography. B and C: Hyperenhancement was illustrated in the arterial phase (B), and the enhancement persists in the portal phase (C). D: A round low density lesion measuring 1.6 x 1.4 cm (white arrow) in the right posterior lobe. The same enhancing pattern was demonstrated in this lesion.

The liver exhibited a small left lobe with uniform density. Two low-density masses measuring 4.0 x 3.7 cm and 1.6 x 1.4 cm were found in the left and right lobes, respectively. The mass in the left lobe was slightly lobulated with mild hyperenhancement in the arterial phase and persistent hyperenhancement in the portal and delayed phase without salient washout (Fig. 1A–C). The other lesion had a demarcated border and presented the same enhancing pattern (Fig. 1 D). The spleen was not absent on CT images. A diagnosis of the hepatic lesions was uncertain based on the CECT findings.

#### 2.1.2 CEUS imaging

Slightly increased echogenicity of the liver was shown on pre-contrast ultrasound scan, consistent with fatty liver. Three hypoechoic FLL were identified in the liver. Two masses in the left lobe were detected with an anterior and posterior layout, measuring 5.3 x 4.5 cm and 2.5 x 2.3 cm, respectively. Another lesion measuring 2.3 x 1.7 cm was observed in the right posterior hepatic lobe. All 3 lesions displayed homogeneous echotexture, demarcated margins, and round shape. Color Doppler revealed a dotted and strip-like blood flow signal inside and around the lesions. Moreover, a hypoechoic oval nodule measuring 1.9 x 1.1 cm was found in the gastro-hepatic ligament area with a clear border and uniform echogenicity (Fig. 2  A–D).

**Figure 2:**
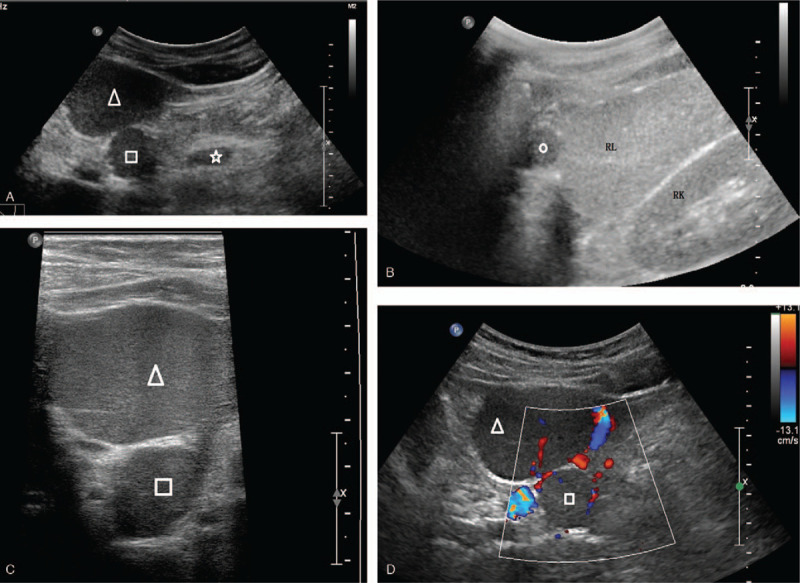
The pre-contrast and contrast-enhanced US scanning of a 55 year old man complaining upper abdominal pain. A: Two hypoechoic focal liver lesions were demonstrated in the left lobe of the liver on pre-contrast US, measuring 5.3 x 4.5 cm (△) and 2.5 x 2.3 cm (□), respectively. The lesions were round in shape with a demarcated border and homogeneous echotexture. B: A smaller lesion measuring 2.3 x 1.7 cm (○) with similar sonographic appearance was seen in the right lobe of the liver. C: Homogeneous echotexture of the lesions was better appreciated on sonogram using linear probe with higher frequency. D: Dotty and strip-like blood flow signal inside and around the lesions demonstrated by color Doppler ultrasound. E-H: Homogenous hyperenhancement of the lesion was illustrated in the arterial phase (E) and portal venous phase (G) and late phase (F). F: The contrast agent in the background liver parenchyma was clearly washed out 3 minutes after the injection compared to the persistent hyperenhancement of the lesions. H: A similar enhancing pattern was observed in the hypoechoic nodule in the gastro-hepatic ligament. Note the prolonged hyperenhancement of the nodule (☆). RK = right kidney, RL = right lobe.

**Figure 2 (Continued):**
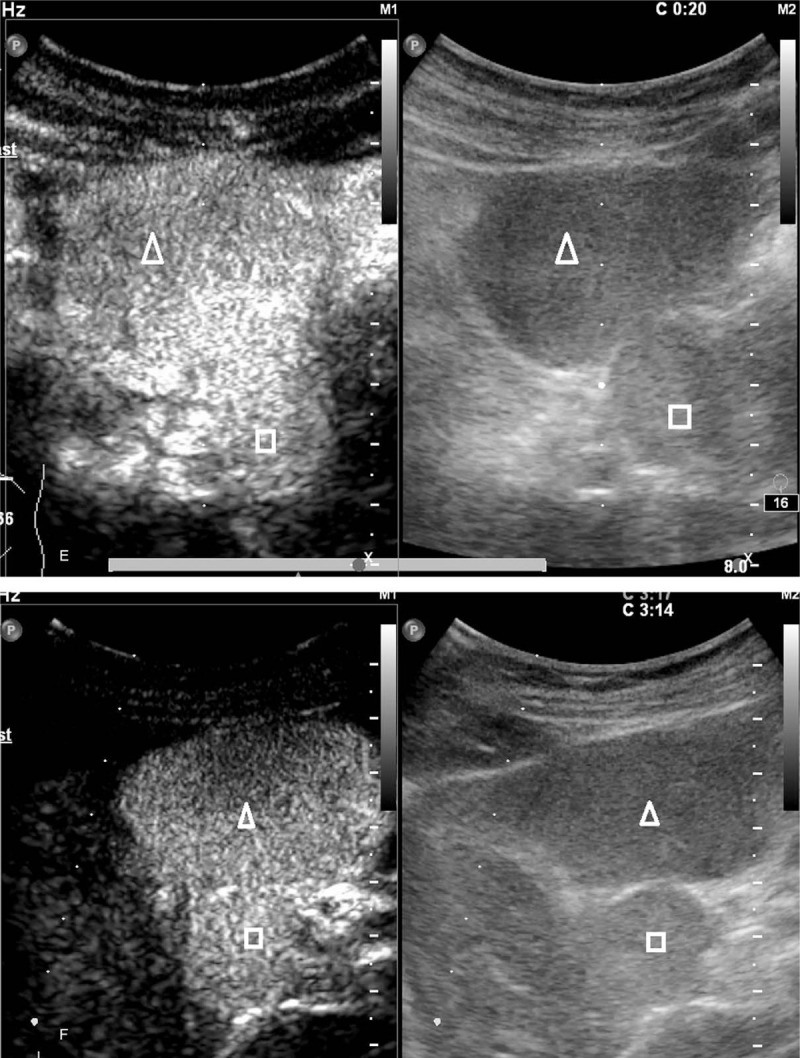
The pre-contrast and contrast-enhanced US scanning of a 55 year old man complaining upper abdominal pain. A: Two hypoechoic focal liver lesions were demonstrated in the left lobe of the liver on pre-contrast US, measuring 5.3 x 4.5 cm (△) and 2.5 x 2.3 cm (□), respectively. The lesions were round in shape with a demarcated border and homogeneous echotexture. B: A smaller lesion measuring 2.3 x 1.7 cm (○) with similar sonographic appearance was seen in the right lobe of the liver. C: Homogeneous echotexture of the lesions was better appreciated on sonogram using linear probe with higher frequency. D: Dotty and strip-like blood flow signal inside and around the lesions demonstrated by color Doppler ultrasound. E-H: Homogenous hyperenhancement of the lesion was illustrated in the arterial phase (E) and portal venous phase (G) and late phase (F). F: The contrast agent in the background liver parenchyma was clearly washed out 3 minutes after the injection compared to the persistent hyperenhancement of the lesions. H: A similar enhancing pattern was observed in the hypoechoic nodule in the gastro-hepatic ligament. Note the prolonged hyperenhancement of the nodule (☆). RK = right kidney, RL = right lobe.

**Figure 2 (Continued):**
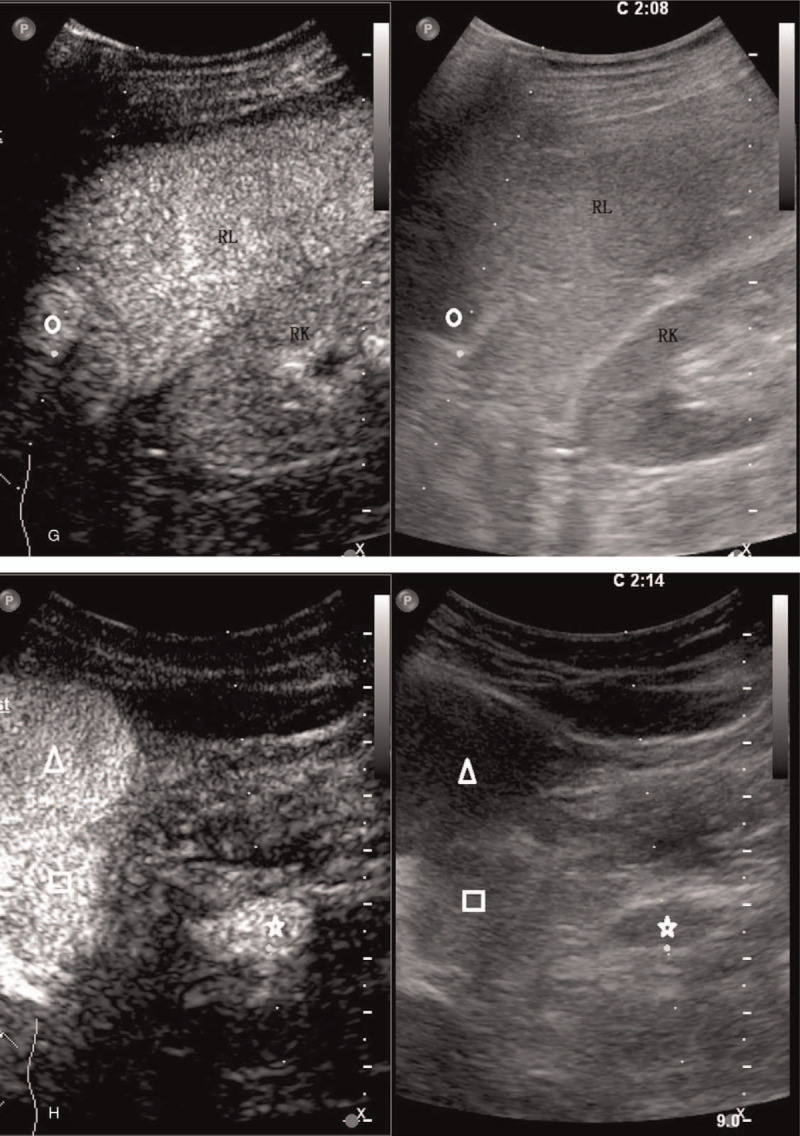
The pre-contrast and contrast-enhanced US scanning of a 55 year old man complaining upper abdominal pain. A: Two hypoechoic focal liver lesions were demonstrated in the left lobe of the liver on pre-contrast US, measuring 5.3 x 4.5 cm (△) and 2.5 x 2.3 cm (□), respectively. The lesions were round in shape with a demarcated border and homogeneous echotexture. B: A smaller lesion measuring 2.3 x 1.7 cm (○) with similar sonographic appearance was seen in the right lobe of the liver. C: Homogeneous echotexture of the lesions was better appreciated on sonogram using linear probe with higher frequency. D: Dotty and strip-like blood flow signal inside and around the lesions demonstrated by color Doppler ultrasound. E-H: Homogenous hyperenhancement of the lesion was illustrated in the arterial phase (E) and portal venous phase (G) and late phase (F). F: The contrast agent in the background liver parenchyma was clearly washed out 3 minutes after the injection compared to the persistent hyperenhancement of the lesions. H: A similar enhancing pattern was observed in the hypoechoic nodule in the gastro-hepatic ligament. Note the prolonged hyperenhancement of the nodule (☆). RK = right kidney, RL = right lobe.

CEUS examination was performed using a Philips iU22 (Philips Medical Solutions; Mountain View, CA) ultrasound system equipped with a C5-1 and L9-3 transducer. Pulse inversion harmonic imaging and mechanical index of less than 0.1 were utilized for CE study. A bolus injection of 1.2 mL SonoVue (Bracco, Milan, Italy) was administered through a 20-gauge catheter line placed in the antecubital vein, followed by a flush of 5 mL 0.9% sodium chloride solution. The imaging timer was started simultaneously with the injection of SonoVue.

The hepatic masses were observed continuously during the first minute and intermittently for the following 4 minutes. All 3 lesions exhibited homogeneous hyperenhancement in the arterial phase without washout in the portal and late phase. The contrast agent in the background liver parenchyma was clearly washed out 3 minutes after the injection compared to the persistent hyperenhancement of the aforementioned lesions (Fig. 2  E–G). A similar enhancing pattern was observed in the hypoechoic nodule in the gastro-hepatic ligament (Fig. 2  H).

All the 3 FLL were suspected to be benign liver tumors according to the findings on CEUS examination. The nodule in the gastro-hepatic ligament area was suspected to be an enlarged lymph node.

### 2.2 Results

**Figure 3:**
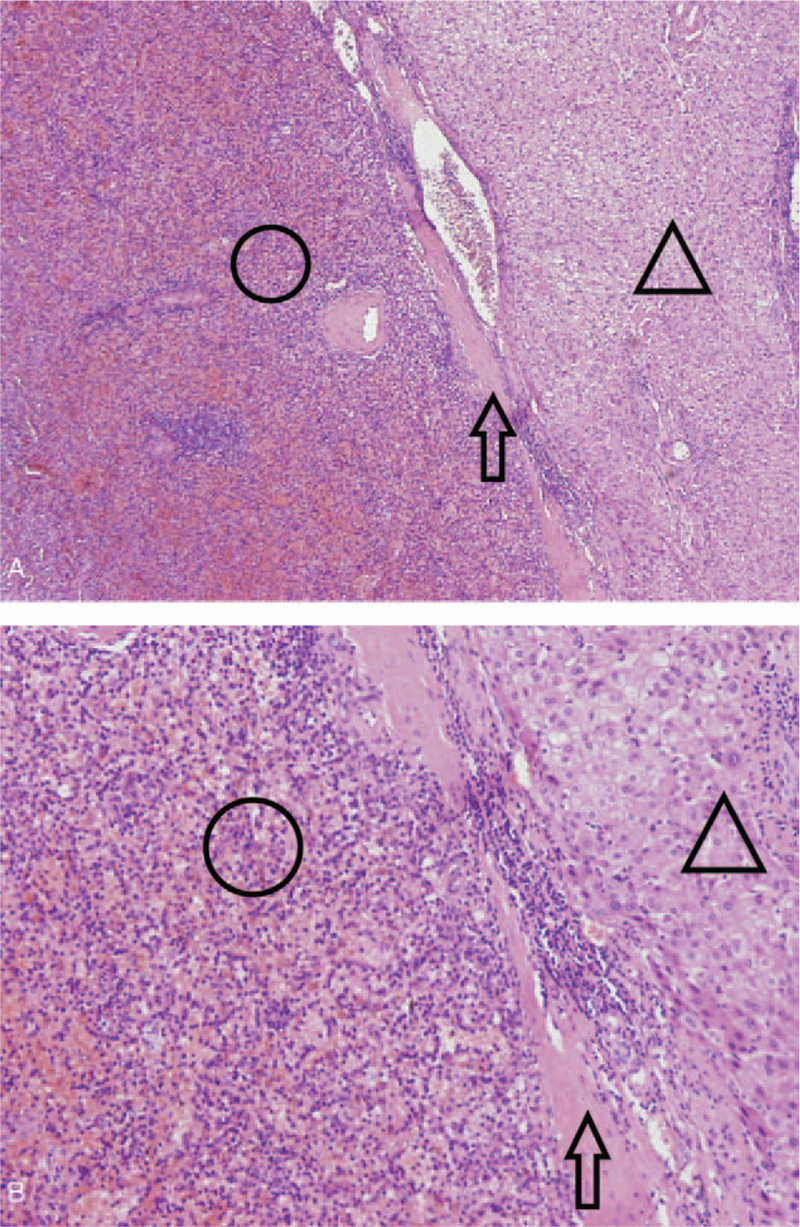
Pathology results of the excised liver tumor in a 55-year-old man. hematoxylin and eosin staining of the paraffin embedded specimen in 40-fold (A) and 100-fold (B) images showing the areas of the spleen (circle) and the liver (triangle) were clearly divided by fibrous connective tissue (arrow). HE = hematoxylin and eosin.

Due to the uncertainty of the diagnosis of multiple liver lesions and suspected lymphadenopathy, laparoscopic abdominal exploration was performed after multidisciplinary discussion and the consensus of the patient. During the surgery, the omentum was found to be adhered to the abdominal wall. Several dark red nodules, ranging from 0.2 to 0.6 cm in size, were observed between the abdominal wall and the diaphragm. The liver texture and color were unremarkable. Two exogenous dark red masses were visible in the left lobe of the liver, measuring 6 x 5 cm and 3 x 2 cm, respectively. A similar mass of 2 cm in diameter was identified between the right lobe the liver and the abdominal wall. Tissue samples from the nodules under the diaphragm were sent for intraoperative pathological examination, which excluded the diagnosis of malignant tumor. Subsequently, the lesions were excised together with the left lateral lobe. The specimens were proven to be splenic tissue by pathology (Fig. 3).

## 3 Discussion

Splenosis usually occurs after spleen trauma or surgery, which may result in direct dissemination of splenic tissue debris. Splenic tissue implants may occur in various parts of the abdominal cavity but are most commonly found in the intestinal serosa, mesentery, and diaphragm. Implantation usually involves multiple lesions, ranging from a few millimeters to 7 cm in diameter, and even greater than 12 cm.^[7]^ Recently, a review of 31 imaging studies of peritoneal splenosis reported that the site frequency of splenosis was greater omentum (51.6%), stomach (25.8%), pancreatic tail (19.4%), left kidney (12.9%), liver (3.2%), and right kidney (3.2%).^[3]^

Surgical exploration is more sensitive compared to imaging for identifying sub-centimeter lesions. In this case, the number of lesions detected by CT and US were less than that found during the operation. Multiple sub-centimeter small nodules in the omentum and peritoneum were missed by both imaging modalities.

Splenic tissue implantation exhibits compensatory enlargement attributed to the recovery of spleen function after splenectomy, which is of certain physiological significance.^[1]^ Therefore, a diagnosis of splenosis is of considerable importance to avoid unnecessary surgery. However, a noninvasive diagnosis of splenosis is challenging since it is prone to mimicking other benign or malignant tumors.

CECT of normal spleen may display a mottled pattern of enhancement in the arterial phase and early portal venous phase due to the different flow rates of the cord and sinuses in the red pulp.^[8]^ However, normal splenic vasculature is absent in the implanted splenic tissue,^[4]^ which may result in different blood supply. The implant splenic tissue is homogenously enhanced without the characteristic mottled pattern observed during the arterial phase on CECT, which makes a diagnosis of hepatic splenosis difficult.

The application of CEUS examination for the diagnosis of FLL has been recommended by many national and international associations.^[9]^ SonoVue is reported as a pure blood pool contrast agent, that is, the microbubbles only remain in the blood vessel for a period of time after the peripheral intravenous injection. The pharmacological kinetics of SonoVue contrast study in 20 healthy adults illustrated that^[10]^ contrast agent intake of both kidneys and right hepatic lobe decreased from 88% to 67% within 5 minutes, but there was almost no such phenomenon in the spleen (90%–99% intake). SonoVue produces a spleen-specific enhancement that lasts for up to 5 minutes, although the mechanism is not yet clear regarding whether the contrast microbubbles are only retained in the spleen parenchyma or phagocytized by macrophages. However, this characteristic CEUS finding has a specific value for the diagnosis of splenosis.

Hyperenhancement in arterial phase can occur in both benign and malignant hepatic neoplasms, such as focal nodular hyperplasia and hepatocellular carcinoma. However, portal venous blood volume of malignant tumors is smaller compared to the normal liver parenchyma, which leads to the washout of contrast agent in the portal and/or late phase. In comparison, benign tumors usually have a larger blood volume. Therefore, enhancement usually persists during the late phase. However, the enhancing time of benign liver tumors is usually not as long as the parenchyma of the spleen, in accordance with the findings of Lim.^[10]^ In addition, the nodule in the gastrohepatic ligament demonstrated similar enhancing pattern as the hepatic lesions in this case. This nodule was also proven to be splenic tissue implantation by pathology. The misdiagnosis of an enlarged lymph node by CEUS examination was due to a lack of experience with extrahepatic application of CEUS examination at that time.

Of note, the left hepatic masses were located next to the ligamentum teres, which is in accordance with cases reported by Gruen DR and Abu Hilal.^[7,11]^ Zekai mentioned that the location of the lesion in the peritoneal folding area may be an important imaging feature for hepatic splenosis.^[12]^ The mechanism of this phenomenon may be due to the direct dissemination of splenic tissue in the peritoneal folding area.

In conclusion, for patients with a history of spleen trauma or surgery, hepatic splenosis should be considered if homogeneous hypoechoic masses are illustrated by US. Homogeneous hyperenhancement of hepatic splenosis on CEUS characteristically persists more than 5 minutes, which is substantially longer than for the normal hepatic parenchyma. Multiple masses with similar enhancing patterns found in other parts of the abdomen can enhance the confidence of a diagnosis of splenosis. Histopathology is the gold standard of hepatic splenosis. However, CEUS can provide valuable imaging information and aid in preoperative diagnosis. As this study is a case report, we hope to further verify these findings in a larger sample size.

## Author contributions

**Data curation:** Xiaofei Zhong, Jiayan Huang.

**Funding acquisition:** Qiang Lu.

**Investigation:** Xiaofei Zhong.

**Methodology:** Qiang Lu.

**Resources:** Lulu Yang, Qiang Lu.

**Software:** Xiaofei Zhong.

**Supervision:** Qiang Lu.

**Visualization:** Lulu Yang, Liping Deng, Ling Nie.

**Writing – original draft:** Xiaofei Zhong.

**Writing – review & editing:** Jiayan Huang, Qiang Lu.
